# Supplementary material for: Single-Cell Analyses Offer Insights into the Different Remodeling Programs of Arteries and Veins
Source: Cells. 2024 May 7;13(10):793. doi: 10.3390/cells13100793 (PMC11119253; doi:10.3390/cells13100793)
Supplement: Supplementary file 1 [file cells-13-00793-s001.zip › Supplementary Methods Cells.pdf]

SUPPLEMENTARY METHODS

Study Subjects and Sample Collection

The study cohort included 30 deceased organ donors whose tissues were donated for research through a collaboration with the Life Alliance Organ Recovery Agency (no informed consent required; **Table S1**). Cross-sectional samples of upper extremity vessels (18 basilic veins, 3 cephalic veins, 24 brachial arteries), approximately 2 cm in length, were obtained *post mortem* following organ procurement procedures. For single-cell RNA sequencing or flow cytometry, vessels were collected in cold DMEM/F12 (Gibco, Thermo Fisher Scientific, Waltham, MA) supplemented with 10% FBS (Sigma-Aldrich, St. Louis, MO), 1 mM sodium pyruvate, 100 U/mL penicillin, 100 ug/mL streptomycin, and 50 ug/mL gentamicin (all Gibco) for tissue dissociation. The remaining samples were collected in RNA*later* (QIAGEN, Germantown, MD) and stored at -80°C. A 5 mm cross-section was fixed in 10% neutral formalin (Sigma-Aldrich, St. Louis, MO) for paraffin embedding and sectioning.

Tissue Dissociation

To generate single-cell suspensions for sequencing or flow cytometry, vessels were cut longitudinally with a sterile scalpel and then into 1-2 mm square sections. Samples were enzymatically digested for 90 minutes at 37°C with shaking using a combination of 3 mg/mL collagenase type II, 0.25 mg/mL soybean trypsin inhibitor, 0.2 mg/mL elastase, 1.4 U/mL Dispase, 60 U/mL DNAse I (all Worthington Biochemical, Lakewood, NJ), 0.15 mg/mL collagenase type XI, 0.25 mg/mL hyaluronidase type I, and 2.38 mg/mL HEPES (all Sigma-Aldrich) in HBSS with Ca^{2+} and Mg^{2+} (Thermo Fisher). After 90 minutes, an equal volume of

Accumax (Sigma-Aldrich) was added to the dissociation mix and incubated for an additional 5 minutes. Single cells were filtered through a 40 µM strainer, washed with HBSS twice, and incubated with red blood cell lysis buffer (BioLegend, San Diego, CA) for 5-10 min. Cells were washed once again with HBSS and resuspended in 0.1% BSA in PBS (Gibco) for counting and RNA sequencing, or in PBS for flow cytometry staining.

Single-Cell RNA Sequencing and Alignment

Preparation of single-cell RNA libraries and sequencing were performed in the Center for Genome Technology at the University of Miami John P. Hussman Institute for Human Genomics. Single cell suspensions were counted using both the Cellometer K2 Fluorescent Viability Cell Counter (Nexcelom) and a hemocytometer. Samples with >80% viability were run using the Chromium Single Cell 3′ Library & Gel Bead Kit v3 (10X Genomics). The manufacturer's protocol was used with a target capture of 10,000 cells. Each sample was processed on an independent Chromium Single Cell A Chip (10X Genomics) and subsequently run on a thermocycler (Eppendorf). Sequencing libraries were evaluated for quality on the Agilent Tape Station (Agilent Technologies, Palo Alto, CA), quantified using a Qubit 2.0 Fluorometer (Invitrogen, Carlsbad, CA), and qPCR before sequencing on the Illumina NovaSeq 6000. FASTQ files were generated with Cell Ranger's mkfastq pipeline (version 6.0.2). The Cell Ranger's count pipeline (version 6.0.2) was used to generate raw gene-barcode matrices from alignment to the 10X Genomics pre-built Cell Ranger human reference package (version 2020-A), from GRCh38 Ensembl build 98/GENCODE v32 gene annotations.

Bioinformatic Quality Control

Data were processed in R (4.2.2) using Seurat v4.[1] Briefly, cells with >15% mitochondrial content, <800 genes (nFeature_RNA), or predicted as doublets according to DoubletFinder[2] were filtered out from downstream analyses. Data were normalized using the "LogNormalize" method and using a scale factor of 10,000. Using Seurat's Scale.Data() function and "vars.to.regress" option, cell cycle, percent mitochondrial genes, and number of UMIs were used to regress out unwanted sources of variation. Unless noted otherwise, all bioinformatic packages were used as detailed in their respective vignettes with no major modifications to the R/Python Code.

Cell Clustering and Functional Annotations

On the merged Seurat object including all 6 vessels from our study, 3 human coronary arteries (GEO accession number GSE131780)[3], and 3 saphenous veins (BioProject accession number PRJNA835590)[4] we applied the functions *NormalizeData*, *FindVariableFeatures*, *ScaleData*, and *RunPCA* using Seurat's standard parameters. We then removed batch effects and generated an integrated map using Harmony.[5] Overall clustering was performed with *FindNeighboors* and the first 30 principal components, followed by *Findclusters* and *RunUMAP* at a 0.5 resolution. We then tested several resolutions (from 0.2 to 1.0, by increments of 0.1) for the *Findclusters* function to find the optimal clustering parameter for separation of modulated SMCs and activated fibroblasts (0.8 in our case).

We manually annotated the main clusters according to canonical cell markers.[6-8] Clusters defined by markers of cell division (*CENPF, MKI67, TOP2A*) or with less than 50 cells were not included in downstream analyses. From a total of 19,475 cells obtained in brachial arteries and

19,167 in basilic/cephalic veins after 10X droplet generation and sequencing, 30,256 high quality cells (arteries: 14,622; veins: 15,634) remained after QC filters and exclusion of dividing cells. Minor cell populations with total cell counts per cluster <250 among all samples analyzed were not included in the final UMAP. These included B cells (70 cells), mast cells (204), neutrophils (91), and Schwann cells (95). A total of 14,360 cells for arteries and 15,355 for veins were plotted in the final UMAP.

From the general Seurat object, the 6 upper arm vessels of interest were subset for downstream bioinformatic analyses. Functional scores were added to the integrated Seurat object using the function *AddModuleScore* in Seurat 4.0. Scores were based on curated gene signature modules from the Molecular Signatures Database (Human MSigDB v2023.1.Hs)[9,10], including the REACTOME modules R-HSA-446353, R-HSA-202733, R-HSA-445355, R-HSA-9707564, R-HSA-1234174, and R-HSA-3299685. Additional scores were generated using the top 200 genes for inflammation in the GeneCards database (version 5.17),[11] the collagens and proteoglycans gene sets from the Matrisome Database,[12] and genes associated with phenotypic modulation of SMCs in atherosclerosis and aneurysmal disease[6,13] [*IGFBP2, CCN2, FN1, KLF4, TGFB1, SERPINE1, COL1A1, TNFRSF11B, VCAM1, MMP2, MGP, NUPR1, ELN, LUM, LAMC3, LCN2, CYTL1, COL8A1, BGN, FMOD, LOXL1, COMP, THBS1, FBLN2, FBLN5, FBN1, TIMP1, LAMA2, TIMP3, TIMP4*]. Heatmaps of module scores were generated using ggplot2. Other heatmaps containing scaled normalized gene expression were generated using the Seurat function *DoHeatmap* and the top 200 significant genes by padj < 0.01 and Log2FC $> |0.5|$. Only genes available in the scale.data slot (top 2,000 most variable genes by Seurat's *FindVariableFeatures)* were displayed in the heatmap. Cell proportions were compared using the propeller method.[14]

Bioinformatic Inference Analyses

Ligand-receptor interactomes were analyzed using CellChat.[15] We imported the clustering metadata and generated a CellChat object with all subclusters following the package vignette. Overrepresented interactions were calculated using *identifyOverExpressedInteractions* and only those present in at least 20 cells per cluster were retained for downstream analysis.

RNA and Protein Extractions

For total RNA isolation, 1-mm cross-sections (~50-60 mg of tissue) of artery and vein pairs from 7 donors were ground to a fine powder in a Spex/Mill 6770 cryogenic grinder (SPEX SamplePrep, Metuchen, NJ) in the presence of 100 uL of Trizol (Thermo Fisher Scientific, Waltham, MA). The grinding program consisted of 15 minutes pre-cooling followed by 15 cycles total of 30 seconds run (10 Hz rate) and 2 minutes cooling per cycle. Total RNA was isolated with Trizol (Thermo Fisher Scientific, Waltham, MA) and further purified using the E.Z.N.A. Total RNA Kit I (Omega Bio-tek, Norcross, GA) as previously described.[16]

Proteins from 6 pairs of arteries and veins were extracted using a sub-fractionation method as previously described.[17] Briefly, ~150 mg of frozen tissue were immersed in ice-cold PBS supplemented with 25 mM EDTA, protease inhibitor cocktail (Roche, Basel, Switzerland), and phosphatase inhibitor mixture (PhosStop, Roche), and cut into 8-10 tiny pieces. The diced tissues were then subjected to three sequential extractions. First, proteins were extracted with a hypertonic saline buffer (0.5 M NaCl, Tris 10 mM, pH 7.5) supplemented with 25 mM EDTA, and protease and phosphatase inhibitors as described above for 4 h at room temperature with gentle shaking (1 ml of buffer per 100 mg tissue; 90 rpm). The extracts, rich in membrane-free intracellular proteins, were collected, desalted using Zeba spin desalting columns (Pierce

Biotechnology, Waltham, MA), mixed with 5 volumes of 100% acetone, and incubated at -20°C for >18h. Proteins were precipitated by centrifugation (16,000 x g for 45 min), dried, and stored at -80°C (**Fraction 1**). The remaining tissues were further incubated with 0.1% SDS supplemented with 25 mM EDTA, and protease and phosphatase inhibitors for 4 h at room temperature with gentle shaking (1 ml per 100 mg tissue; 90 rpm). The extracts, enriched in membrane-associated proteins, were collected, and stored at -80°C (**Fraction 2**). The last fraction was obtained by cryo-grinding the remaining material using the Spex/Mill 6770 cryogenic mill (SPEX SamplePrep) in the presence of a chaotropic buffer (4 M guanidine-HCl, 50 mM sodium acetate, pH 5.8, supplemented with EDTA 25 mM, and protease and phosphatase inhibitors). Proteins were further extracted with more chaotropic buffer (0.5 ml per mg original tissue weight) for 48 h at room temperature with vigorous shaking (300 rpm). The extracts, rich in mature ECM proteins, were then collected, mixed with 5 volumes of 100% ethanol, and incubated at -20°C for >18 h. Proteins were precipitated by centrifugation (16,000 x g for 45 min), washed with 90% ethanol, dried, and stored at -80°C (**Fraction 3**). Dried proteins from the first and third fractions were reconstituted in 8M urea supplemented with 25 mM EDTA. Total proteins from the three fractions were assayed using BCA method (Pierce Biotechnology).

Bulk RNA Sequencing and Bioinformatic Analyses

Preparation and sequencing of RNA libraries was carried out in the Center for Genome Technology at the University of Miami John P. Hussman Institute for Human Genomics. Briefly, total RNA from 7 pairs of arteries and veins was quantified and qualified using the Agilent Bioanalyzer to have an RNA Integrity Score (RIN)>5. 500 ng of total RNA was used as input for the Illumina TruSeq Stranded Total RNA Library Prep Kit with Ribo-Zero to create ribosomal

RNA-depleted sequencing libraries. Each sample had a unique barcode to allow for multiplexing and was sequenced to >40 million raw reads in a single end 75bp sequencing run on the Illumina NextSeq500. Raw sequence data was processed by the on-instrument Real Time Analysis software (v.2.4.11) to basecall files. These were converted to de-multiplexed FASTQ files with the Illumina supplied scripts in the BCL2FASTQ software (v2.17). The quality of the reads was determined with the FASTQC software (http://www.bioinformatics.babraham.ac.uk/projects/fastqc/) to evaluate per base sequence quality, duplication rates, and overrepresented k-mers. Illumina adapters were trimmed from the ends of the reads using the Trim Galore! package (http://www.bioinformatics.babraham.ac.uk/projects/trim_galore/). Reads were aligned to the human reference genome (hg38) with the STAR aligner (v2.5.2).[18] Gene count quantification for total RNA was performed using the GeneCounts function within STAR against the GENCODE v35 human transcript .gtf file. Differential expressed genes (DEGs) between arteries and veins were identified in DESeq2[19] using a paired algorithm accounting for the donor of each set of paired samples (design= ~Subject + VesselType). The false discovery rate (FDR) was determined by applying the Benjamini-Hochberg multiplicity correction method. Annotation of DEGs in bulk samples as EC-, SMC-, fibroblast-, or immune cell-enriched was based on a log2FC >0.1 and p <0.01 by Wilcoxon rank-sum test using the integrated single-cell map of arteries and veins as reference and the *FindAllMarkers* function in Seurat.

Deconvolution of Bulk RNA-seq Data

Digital cytometry of the 7 sequenced pairs of arteries and veins was performed using the deconvolution method SCDC.[20] The integrated artery and vein map was used as the single cell

reference (*sc.eset* in SCDC) and the bulk RNA-seq unnormalized counts were converted to *bulk.eset* as indicated in the SCDC vignette. We then applied the function getPropBox to infer the coarse cellular proportions in the bulk RNA-seq data.

ECM-Targeted Proteomics

Extracellular matrix-enriched protein aliquots (see **Fraction 3** above) from 3 pairs of arteries and veins were profiled by mass spectrometry at MS Bioworks (Ann Arbor, MI). Briefly, the protein concentration of each sample was determined by Qubit fluorometry (Invitrogen), and 10 ug per sample was processed by SDS-PAGE using a 10% NuPAGE Bis-Tris mini gel (Invitrogen) with the MES buffer system. The gel was run 2 cm and the motility region excised into 10 equally sized bands. Each band was processed by in-gel digestion with trypsin using a robot (DigestPro, CEM, Charlotte, NC) and the following protocol: wash with 25 mM ammonium bicarbonate followed by acetonitrile; reduction with 10 mM dithiothreitol at 60°C followed by alkylation with 50 mM iodoacetamide at room temperature; digestion with sequencing grade trypsin (Promega, Madison, WI) at 37°C for 4 h; and quenching with formic acid. Without further processing, half the supernatant of each digested sample was analyzed directly by nano LC-MS/MS with a Waters M-Class LC system (Milford, MA) interfaced to a Thermo Fisher Exploris 480 mass spectrometer. Peptides were loaded on a trapping column and eluted over a 75 μm analytical column at 350 nL/min. The column was heated to 55°C using a column heater (Sonation GmbH, Biberach, Germany). The mass spectrometer was operated in data-dependent mode for 5 h, with the Orbitrap operating at 60,000 FWHM and 15,000 FWHM for MS and MS/MS, respectively, and 3s cycles for both.

Processing and Analysis of Proteomics Data

Data were searched using a local copy of Mascot (Matrix Science, Boston, MA) with the following parameters: 1) enzyme: trypsin/P; 2) database: SwissProt Human, concatenated forward and reverse plus common contaminants; 3) fixed modifications: carbamidomethyl (C); 4) variable modifications: oxidation (M/P), acetyl (N-term), pyro-glu (N-term Q), deamidation (N, Q); 5) mass values: monoisotopic; 6) peptide mass tolerance: 10 ppm; 7) fragment mass tolerance: 0.02 Da; 8) max missed cleavages: 2. Mascot DAT files were parsed into Scaffold (Proteome Software, Portland, OR) for validation, filtering, and to create a non-redundant list per sample. Data were filtered using a 1% protein and peptide FDR and requiring at least two unique peptides per protein. After removing contaminants, spectral counts (SpC) were converted to Normalized Spectral Abundance Factor (NSAF) using the formula: $NSAF = (SpC/MW)/\Sigma(SpC/MW)_N$, where MW is the protein molecular weight in kDa and N is the total number of proteins detected per sample. Due to the selective enrichment during protein isolation, only proteins classified as ECM[21] and detected in at least two samples were considered for differential expression analysis. To avoid zero values during log2 normalization, proteins with zero counts were assigned a fraction of a spectral count (0.7). Arteries and veins were compared using paired t-tests and differences were considered significant if $\log_2 FC > 1$ and $p < 0.05$.

Flow Cytometry

Quantification of CD90/F2R double-positive myofibroblasts in 3 pairs of arteries and veins was performed by flow cytometry. Cell suspensions from fresh vessels were prepared as indicated above, resuspended in PBS, and counted. An aliquot of cells was incubated with 1 μL of the LIVE/DEAD Fixable Dead Stain Kit (Invitrogen) at 4°C for 30 min. The rest of the

sample was resuspended in FACS buffer (PBS supplemented with 2% FBS, 1 mM EDTA, 0.1% sodium azide) and incubated with 8 μL of Human BD Fc Block (BD Biosciences, Franklin Lakes, NJ) at 4°C for 10 min. After washing and aliquoting into individual flow tubes, cells were labeled for 30 min with 2 μL of the target antibodies (CD90 PE-Cy7, BD Biosciences cat. #561558; PAR1 Alexa fluor 488, R&D Systems, Minneapolis, MN, cat. # fab3855), including single-stain and no-stain controls. All tubes were washed with FACS buffer, followed by fixation with 2% PFA in PBS for 15 min at room temperature. Cells were washed again and stored at 4°C in FACS buffer overnight. Samples were read in a BD LSRFortessa Cell Analyzer and analyzed using FlowJo Software v10.10 (BD Biosciences).

Western Blot

Expression differences in SMC-derived proteins between arteries and veins were validated by WB. For this, 10 μg of Fraction 1 were loaded onto 12% or 4-20% Tris-Glycine SDS-polyacrylamide gels and subsequently transferred to PVDF membranes (GE Healthcare, Marlborough, MA). Specific proteins were detected using antibodies against IGFBP2 (cat. #3922, 1:1000; Cell Signaling Technology, Danvers, MA), and GAPDH (cat. #2118, 1:3000; Cell Signaling Technology). Bound antibodies were detected after sequentially incubating the membranes with HRP-conjugated secondary antibodies, and WesternBright ECL Substrate (Advansta, San Jose, CA) was used for signal detection. Images were captured using the ChemiDocTM-MP Imaging System (BioRad, Hercules, CA). Signal quantification was performed using ImageJ (National Institutes of Health, Bethesda, MD). Expression levels of IGFBP2 were normalized with respect to GAPDH.

Histology and Immunostaining

Tissue sections were stained with Masson's trichrome (#25088-1, Polysciences, Warrington, PA) and Movat's pentachrome (#ab245884, Abcam, Waltham, MA) for gross histomorphometric analysis. Intimal hyperplasia (defined as intimal thickness and intima/intima-media [I/I-M] thickness ratio), medial SMC content (expressed as % area), and medial ECM content (% area) were quantified using ImageJ (National Institutes of Health) and color thresholding methods. The average intimal thickness and I/I-M thickness ratio was calculated from 4 representative measurements of each cross-section.

For immunohistochemistry, paraffin sections were rehydrated by serially immersing them in xylene, alcohol, and water, and antigens retrieved by boiling slides in 10 mM citrate buffer, pH 6.0 or Tris-EDTA buffer, pH 9.0 for 30 minutes (see table below). Sections were incubated with DAKO Peroxidase Blocking Solution (#K4005, Agilent, Santa Clara, CA) for 10 min, followed by DAKO Protein Blocking Solution (#X0909) for 1 hour. Specific proteins were detected by incubating overnight at 4°C with primary antibodies and dilutions as indicated below. Bound antibodies were detected using the DAKO EnVision System HRP labeled polymer anti-rabbit (#K4003) or anti-mouse (#K4001) secondary antibodies, or donkey anti-sheep HRP secondary (1:200, #2473, Santa Cruz biotechnology, Dallas Tx). Color was developed with the Abcam AEC Substrate Kit (#ab64252, Abcam, Boston, MA). Nuclei were counterstained with Gill 3 hematoxylin and mounted in VectaMount AQ Mounting Medium (#H-5501, Vector Laboratories). Images were acquired using a VisionTek DM01 digital microscope (Sakura Finetek, Torrance, CA).

For immunofluorescence, after antigen retrieval as above, sections were treated with 3% hydrogen peroxide and DAKO Protein Blocking Solution (#X0909), followed by primary

antibodies diluted in DAKO Antibody Diluent Solution (#S3022) overnight at 4ºC. Bound antibodies were detected with Alexa Fluor 546 goat anti-rabbit antibody (1:1000, #A11081; all secondary antibodies from Thermo Fisher Scientific), Alexa Fluor 633 goat anti-mouse antibody (1:1000, #A21052), or Alexa Fluor 633 goat anti-rabbit antibody (1:1000, #A21071) for 45 minutes. Sections were counter-stained with 300 nM DAPI solution (#D1306, Thermo Fisher Scientific) in PBS for 5 minutes, and mounted in DABCO antifading polyvinyl alcohol mounting medium (#10981, Sigma-Aldrich). For signal amplification, after overnight incubation with primary antibodies, slides were incubated with biotinylated swine anti-rabbit polyclonal antibodies (1:1000, #E0353, DAKO) or biotinylated goat anti-mouse (1:1000, #OS02B, Oncogene Research) for 1 hour, then streptavidin HRP (1:1000, #P0397, DAKO) for 30 minutes, and finally amplified with a Tyramide Signal Amplification kit (1:50, #NEL700A001KT, Perkin Elmer, Waltham, MA). Amplification of the biotinylated antibody was detected through incubation with a streptavidin conjugated Alexa 546 secondary (1:1000, #S11225, Thermo Fisher Scientific) for 1 hour. Sections were examined in a Keyence All-in-One Fluorescence Microscope BZ-X800L and photographed using the Keyence BZ-X800 Viewer software (Keyence, Itasca, IL).

Statistical Analysis

Statistical analyses of morphometry and protein validation data were performed using GraphPad Prism 8.4.0 (San Diego, CA). Normally distributed data were compared using t-tests and expressed as mean ± standard deviation (SD) or mean ± standard error of the mean (SEM) where indicated. If normality assumptions were not met, the Mann-Whitney test was used, and data expressed as median and interquartile range (IQR). Comparisons of paired arteries and veins

from the same donor were performed using paired t-tests or paired Wilcoxon signed-rank tests as appropriate.

TARGET	ANTIBODY	DILUTION	ANTIGEN RETRIEVAL
CNN1	Abcam, ab46794	1:50, IF	pH 9
COL8A1	Abcam, ab236653	1:50, IHC	pH 9
ITGA10	Antibodies.com, A263375	1:100, IHC	pH 9
ITLN1	Invitrogen, PA5-47601	1:50, IHC	pH 9
PDGFRA	Origene, TA804956	1:100, IF	pH 9
PECAM1	DAKO, M0823	1:50, IF	pH 9

REFERENCES

1. Hao Y, Hao S, Andersen-Nissen E, Mauck WM, 3rd, Zheng S, Butler A, Lee MJ, Wilk AJ, Darby C, Zager M, et al. Integrated analysis of multimodal single-cell data. *Cell*. 2021;184:3573-3587 e3529. doi: 10.1016/j.cell.2021.04.048
2. McGinnis CS, Murrow LM, Gartner ZJ. DoubletFinder: Doublet Detection in Single-Cell RNA Sequencing Data Using Artificial Nearest Neighbors. *Cell Syst*. 2019;8:329-337 e324. doi: 10.1016/j.cels.2019.03.003
3. Wirka RC, Wagh D, Paik DT, Pjanic M, Nguyen T, Miller CL, Kundu R, Nagao M, Coller J, Koyano TK, et al. Atheroprotective roles of smooth muscle cell phenotypic modulation and the TCF21 disease gene as revealed by single-cell analysis. *Nat Med*. 2019;25:1280-1289. doi: 10.1038/s41591-019-0512-5
4. Sun Y, Hu X, Zhang K, Rao M, Yin P, Dong R. A Single-Cell Survey of Cellular Heterogeneity in Human Great Saphenous Veins. *Cells*. 2022;11. doi: 10.3390/cells11172711
5. Korsunsky I, Millard N, Fan J, Slowikowski K, Zhang F, Wei K, Baglaenko Y, Brenner M, Loh PR, Raychaudhuri S. Fast, sensitive and accurate integration of single-cell data with Harmony. *Nat Methods*. 2019;16:1289-1296. doi: 10.1038/s41592-019-0619-0
6. Pedroza AJ, Tashima Y, Shad R, Cheng P, Wirka R, Churovich S, Nakamura K, Yokoyama N, Cui JZ, Iosef C, et al. Single-Cell Transcriptomic Profiling of Vascular Smooth Muscle Cell Phenotype Modulation in Marfan Syndrome Aortic Aneurysm. *Arterioscler Thromb Vasc Biol*. 2020;40:2195-2211. doi: 10.1161/ATVBAHA.120.314670

7. Hu Z, Liu W, Hua X, Chen X, Chang Y, Hu Y, Xu Z, Song J. Single-Cell Transcriptomic Atlas of Different Human Cardiac Arteries Identifies Cell Types Associated With Vascular Physiology. *Arterioscler Thromb Vasc Biol*. 2021;41:1408-1427. doi: 10.1161/ATVBAHA.120.315373
8. Rojas MG, Zigmond ZM, Pereira-Simon S, Santos Falcon N, Suresh Kumar M, Stoyell-Conti FF, Kosanovic C, Griswold AJ, Salama A, Yang X, et al. The intricate cellular ecosystem of human peripheral veins as revealed by single-cell transcriptomic analysis. *PloS one*. 2024;19:e0296264. doi: 10.1371/journal.pone.0296264
9. Subramanian A, Tamayo P, Mootha VK, Mukherjee S, Ebert BL, Gillette MA, Paulovich A, Pomeroy SL, Golub TR, Lander ES, et al. Gene set enrichment analysis: a knowledge-based approach for interpreting genome-wide expression profiles. *Proc Natl Acad Sci U S A*. 2005;102:15545-15550. doi: 10.1073/pnas.0506580102
10. Liberzon A, Birger C, Thorvaldsdottir H, Ghandi M, Mesirov JP, Tamayo P. The Molecular Signatures Database (MSigDB) hallmark gene set collection. *Cell Syst*. 2015;1:417-425. doi: 10.1016/j.cels.2015.12.004
11. Stelzer G, Rosen N, Plaschkes I, Zimmerman S, Twik M, Fishilevich S, Stein TI, Nudel R, Lieder I, Mazor Y, et al. The GeneCards Suite: From Gene Data Mining to Disease Genome Sequence Analyses. *Curr Protoc Bioinformatics*. 2016;54:1 30 31-31 30 33. doi: 10.1002/cpbi.5
12. Shao X, Gomez CD, Kapoor N, Considine JM, Grams C, Gao YT, Naba A. MatrisomeDB 2.0: 2023 updates to the ECM-protein knowledge database. *Nucleic Acids Res*. 2023;51:D1519-D1530. doi: 10.1093/nar/gkac1009
13. Kim J, Procknow JD, Yanagisawa H, Wagenseil JE. Differences in genetic signaling, and not mechanical properties of the wall, are linked to ascending aortic aneurysms in fibulin-4 knockout mice. *Am J Physiol Heart Circ Physiol*. 2015;309:H103-113. doi: 10.1152/ajpheart.00178.2015
14. Phipson B, Sim CB, Porrello ER, Hewitt AW, Powell J, Oshlack A. propeller: testing for differences in cell type proportions in single cell data. *Bioinformatics*. 2022;38:4720-4726. doi: 10.1093/bioinformatics/btac582
15. Jin S, Guerrero-Juarez CF, Zhang L, Chang I, Ramos R, Kuan CH, Myung P, Plikus MV, Nie Q. Inference and analysis of cell-cell communication using CellChat. *Nat Commun*. 2021;12:1088. doi: 10.1038/s41467-021-21246-9
16. Martinez L, Rojas MG, Tabbara M, Pereira-Simon S, Santos Falcon N, Rauf MA, Challa A, Zigmond ZM, Griswold AJ, Duque JC, et al. The Transcriptomics of the Human Vein Transformation After Arteriovenous Fistula Anastomosis Uncovers Layer-Specific Remodeling and Hallmarks of Maturation Failure. *Kidney Int Rep*. 2023;8:837-850. doi: 10.1016/j.ekir.2023.01.008
17. Didangelos A, Yin X, Mandal K, Saje A, Smith A, Xu Q, Jahangiri M, Mayr M. Extracellular matrix composition and remodeling in human abdominal aortic aneurysms: a proteomics approach. *Mol Cell Proteomics*. 2011;10:M111 008128. doi: 10.1074/mcp.M111.008128
18. Dobin A, Davis CA, Schlesinger F, Drenkow J, Zaleski C, Jha S, Batut P, Chaisson M, Gingeras TR. STAR: ultrafast universal RNA-seq aligner. *Bioinformatics*. 2013;29:15-21. doi: 10.1093/bioinformatics/bts635
19. Love MI, Huber W, Anders S. Moderated estimation of fold change and dispersion for RNA-seq data with DESeq2. *Genome Biol*. 2014;15:550. doi: 10.1186/s13059-014-0550-8
20. Dong M, Thennavan A, Urrutia E, Li Y, Perou CM, Zou F, Jiang Y. SCDC: bulk gene expression deconvolution by multiple single-cell RNA sequencing references. *Brief Bioinform*. 2021;22:416-427. doi: 10.1093/bib/bbz166
21. UniProt C. UniProt: the Universal Protein Knowledgebase in 2023. *Nucleic Acids Res*. 2023;51:D523-D531. doi: 10.1093/nar/gkac1052
