# Supplementary figures and images for: Single-Cell Analyses Offer Insights into the Different Remodeling Programs of Arteries and Veins
Source: Cells. 2024 May 7;13(10):793. doi: 10.3390/cells13100793 (PMC11119253; doi:10.3390/cells13100793)

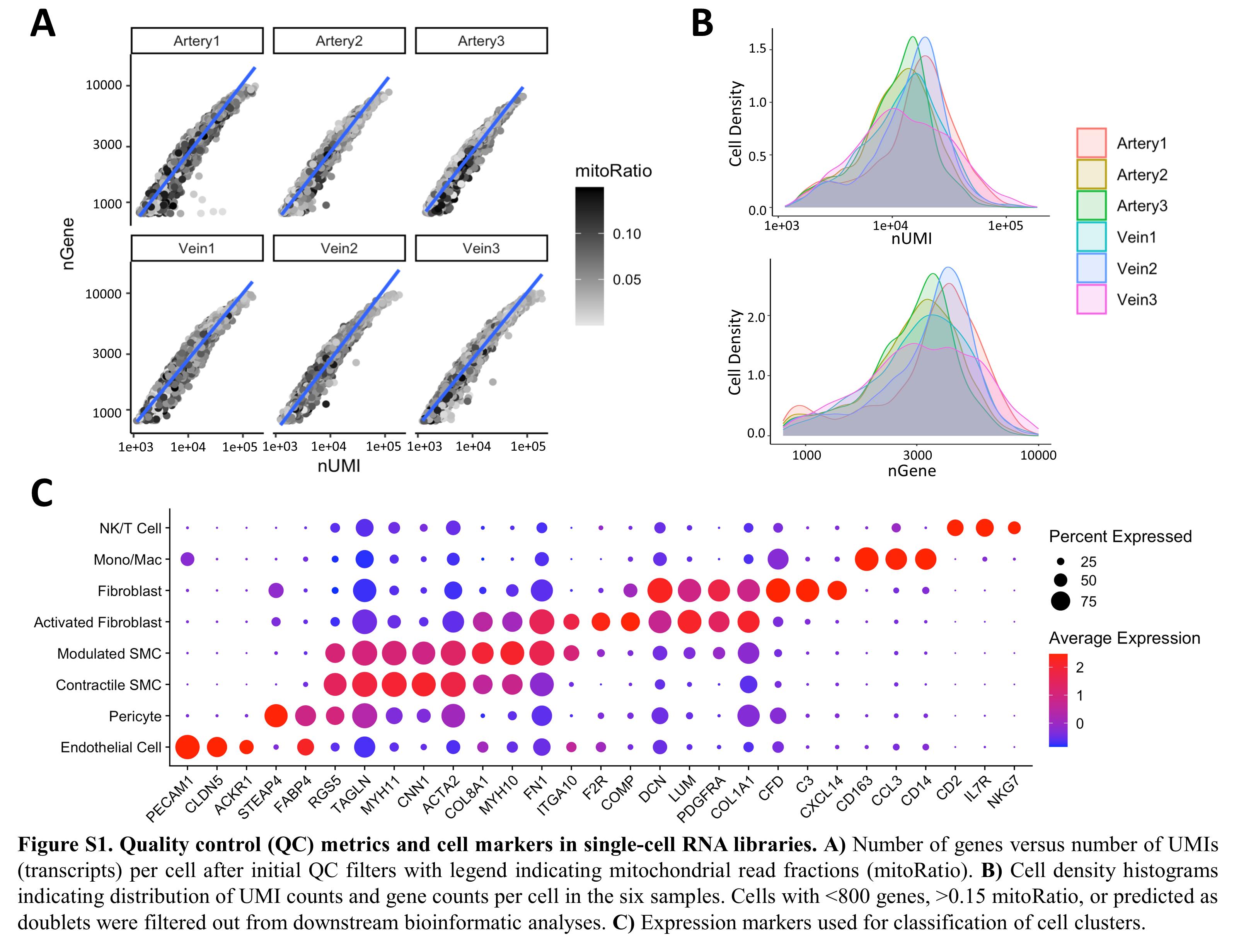

Supplement: Supplementary file 1 [file cells-13-00793-s001.zip › Figure S1.PNG]

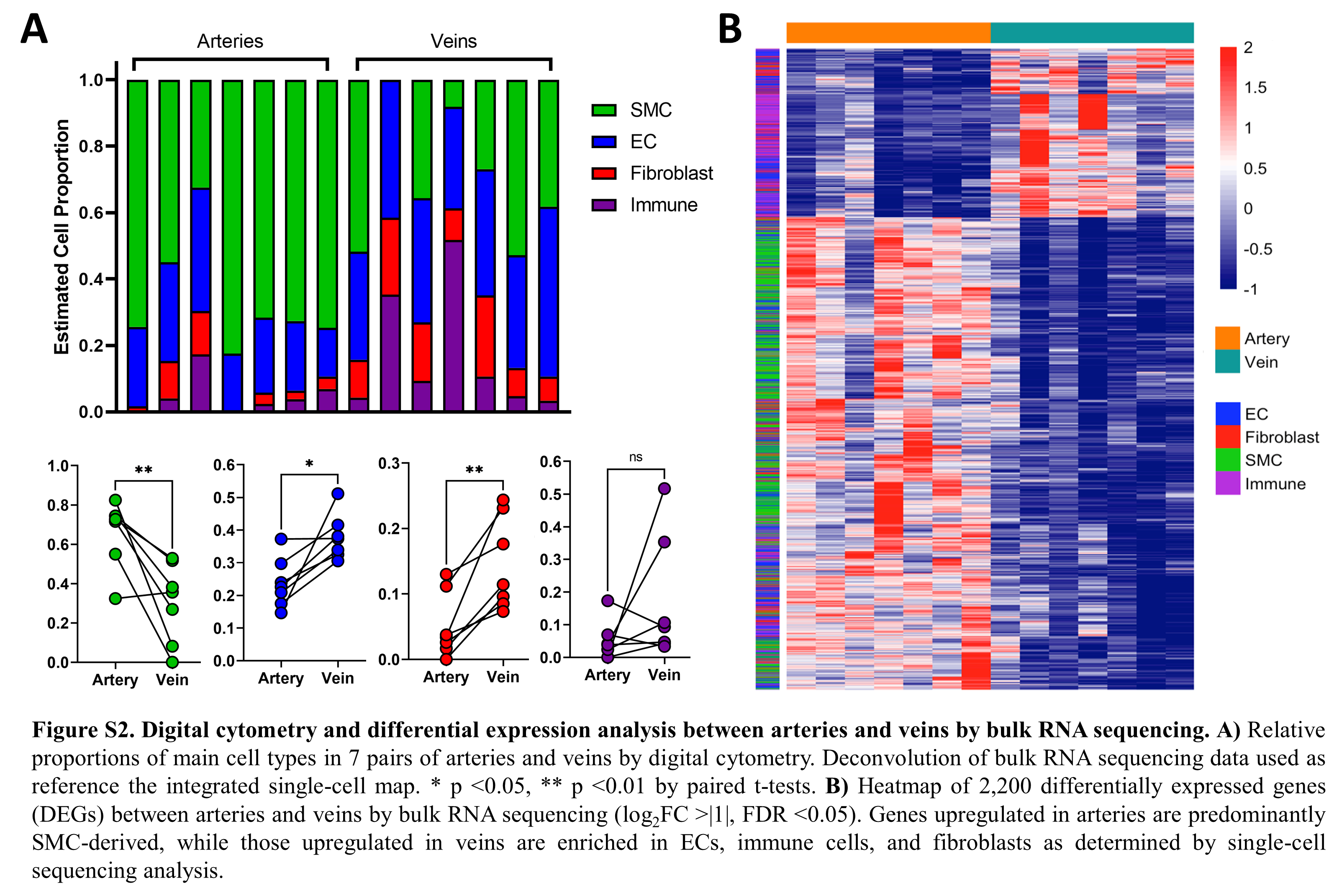

Supplement: Supplementary file 1 [file cells-13-00793-s001.zip › Figure S2.PNG]

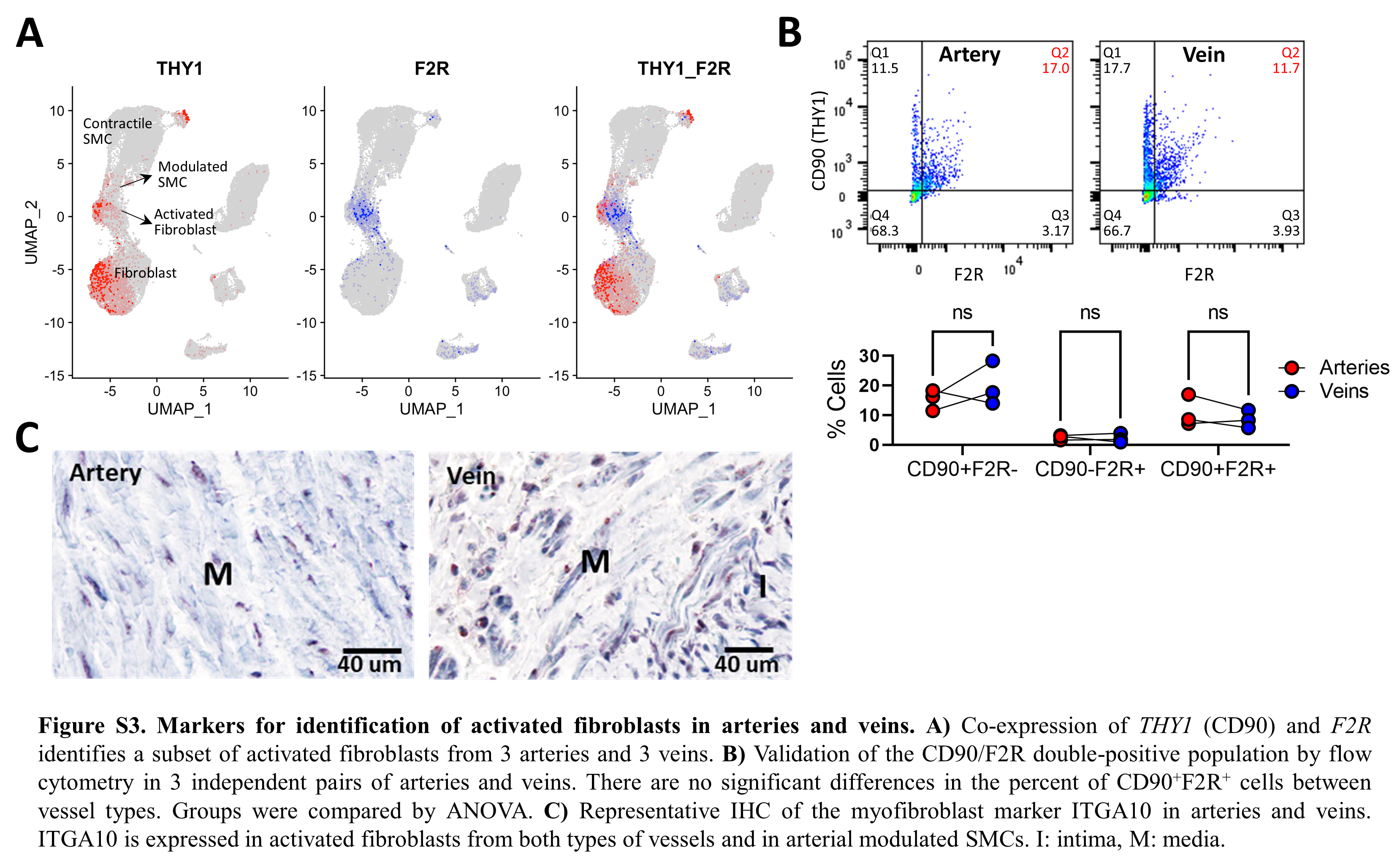

Supplement: Supplementary file 1 [file cells-13-00793-s001.zip › Figure S3.PNG]

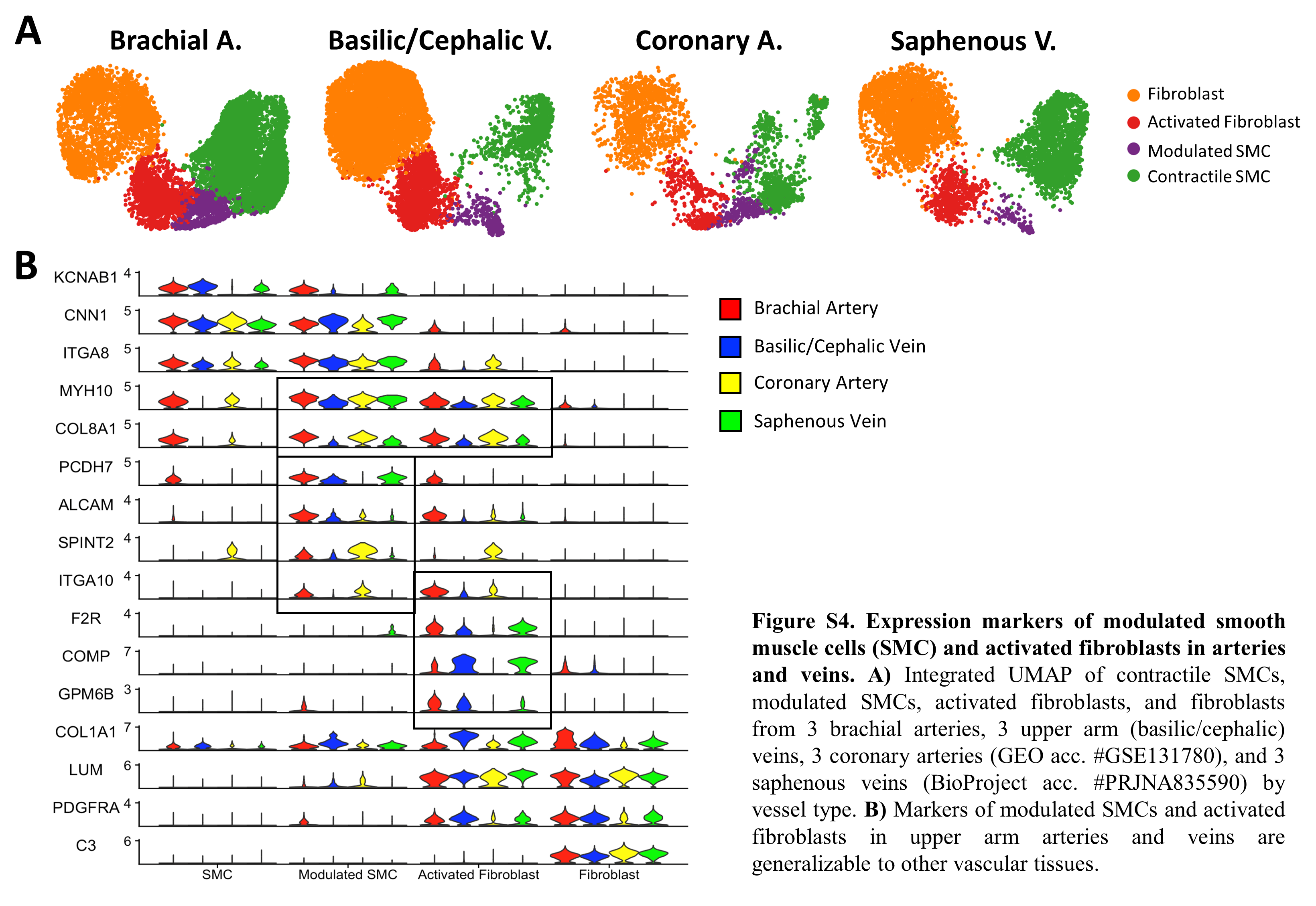

Supplement: Supplementary file 1 [file cells-13-00793-s001.zip › Figure S4.PNG]

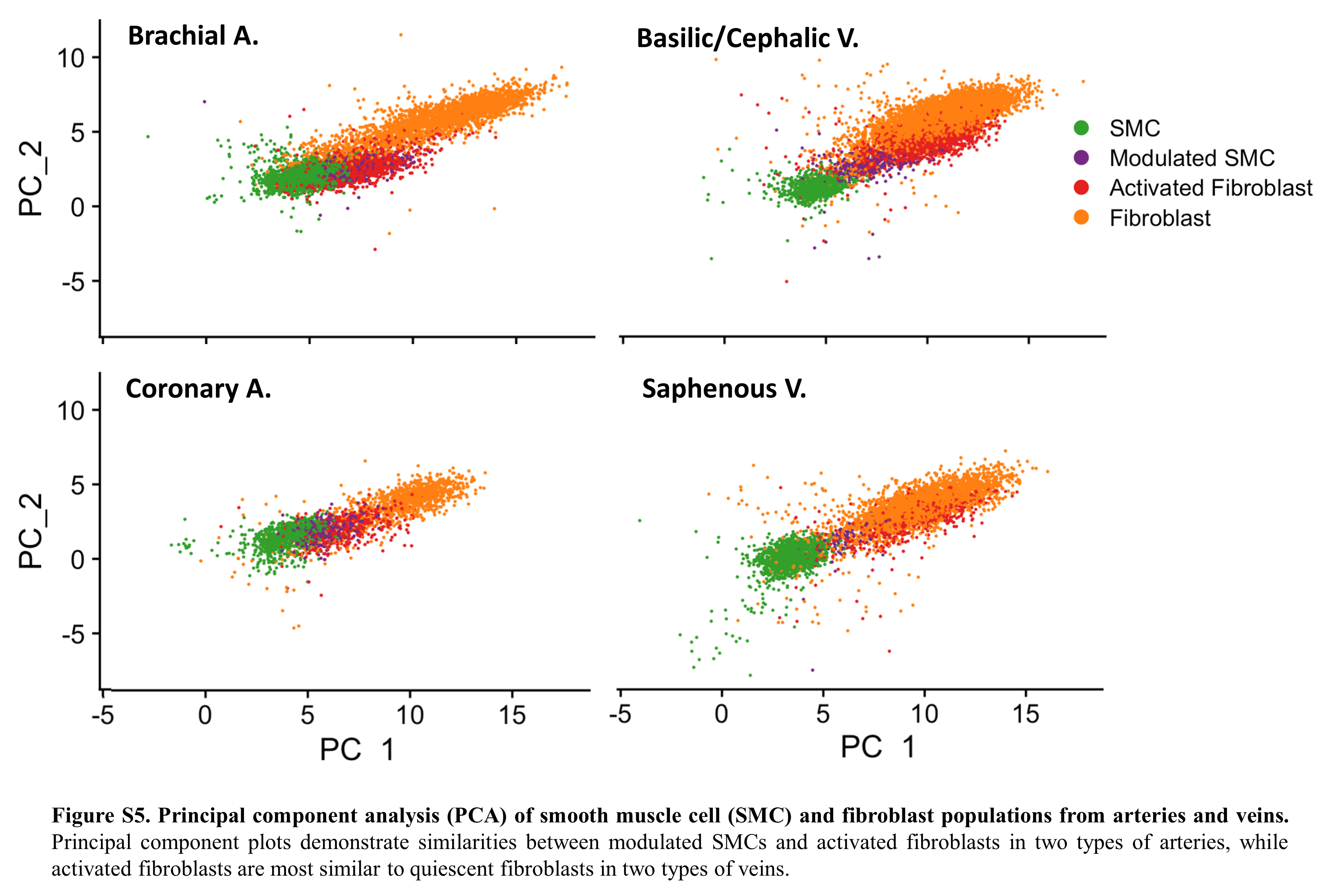

Supplement: Supplementary file 1 [file cells-13-00793-s001.zip › Figure S5.PNG]

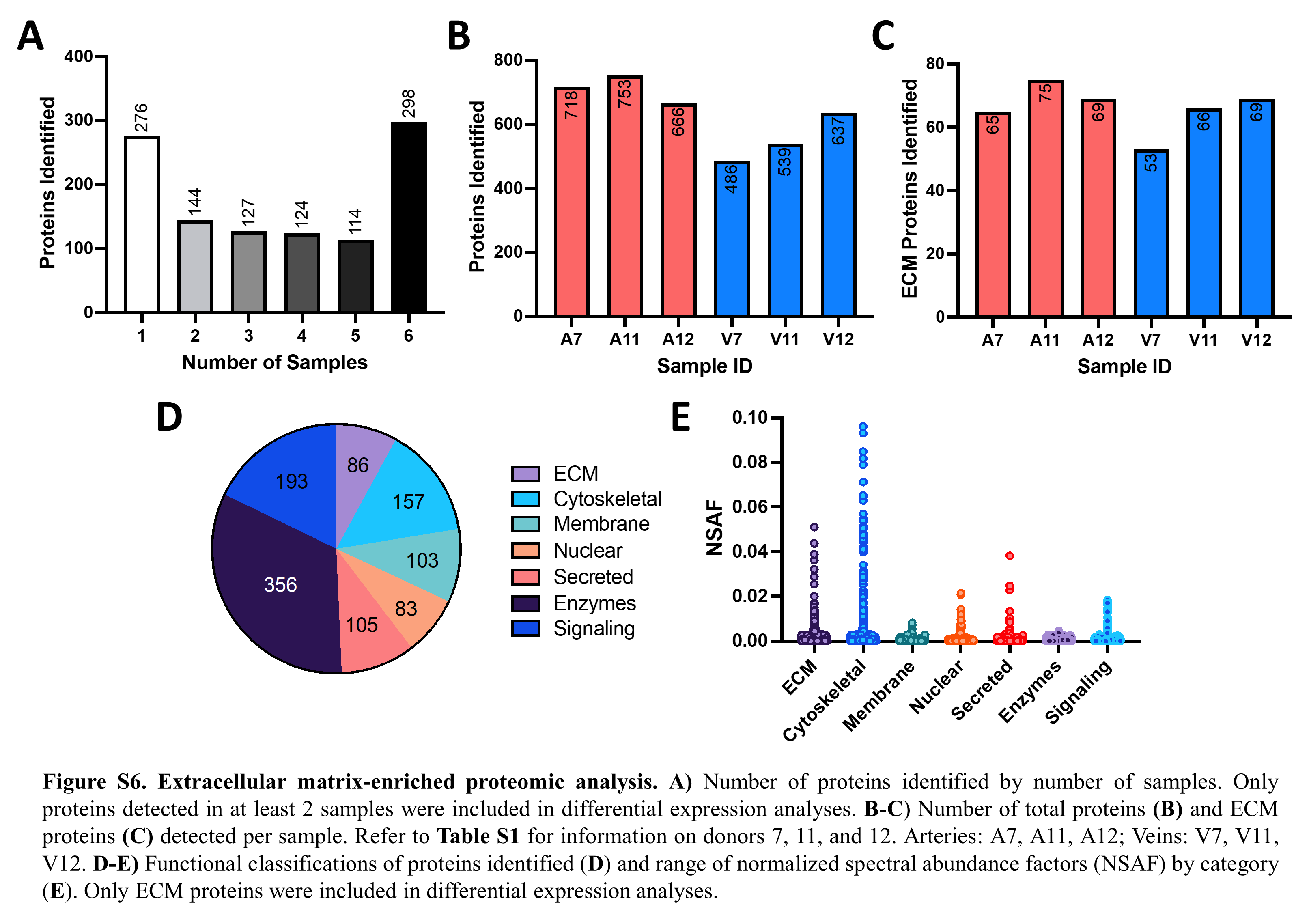

Supplement: Supplementary file 1 [file cells-13-00793-s001.zip › Figure S6.PNG]

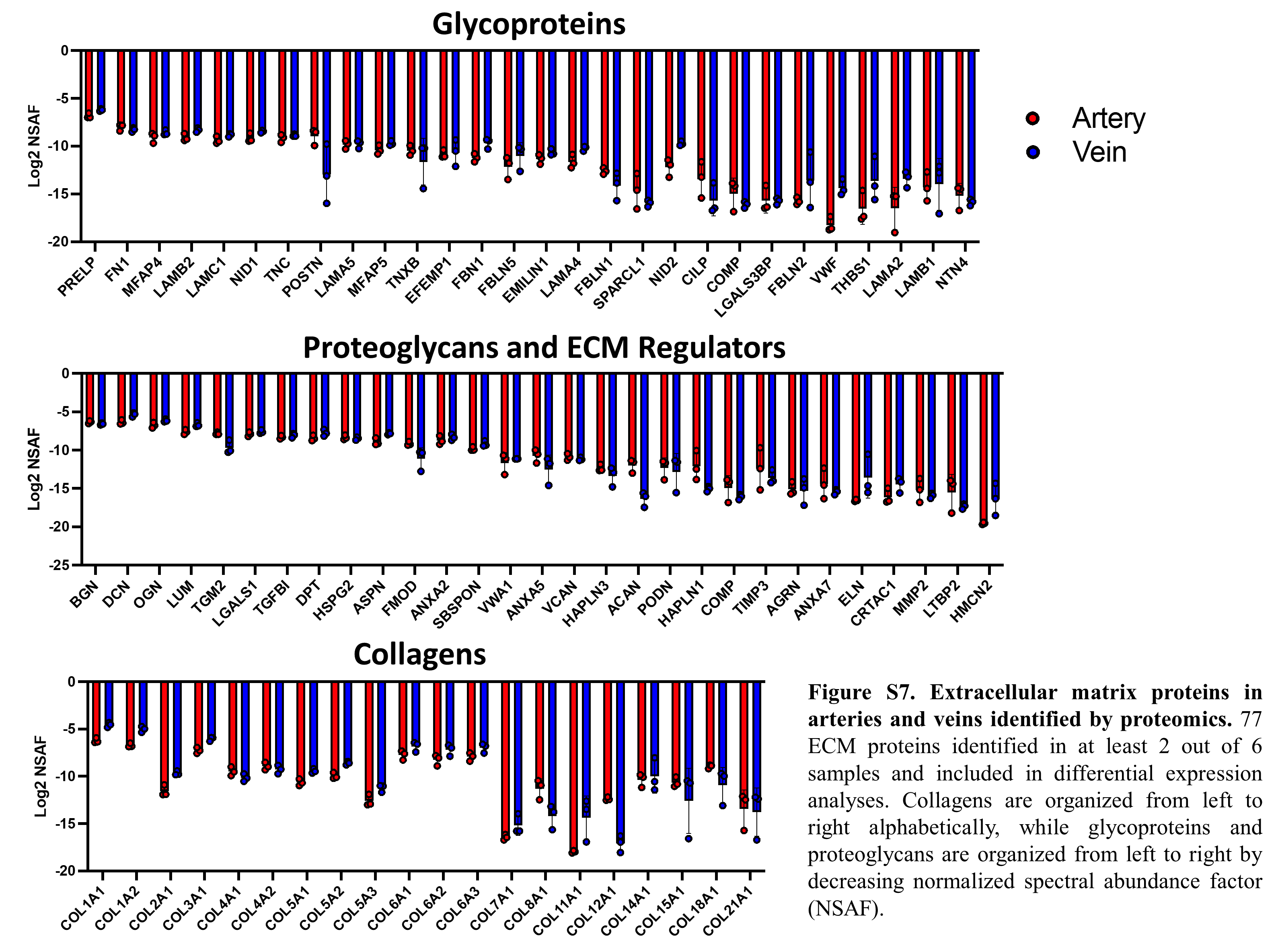

Supplement: Supplementary file 1 [file cells-13-00793-s001.zip › Figure S7.PNG]

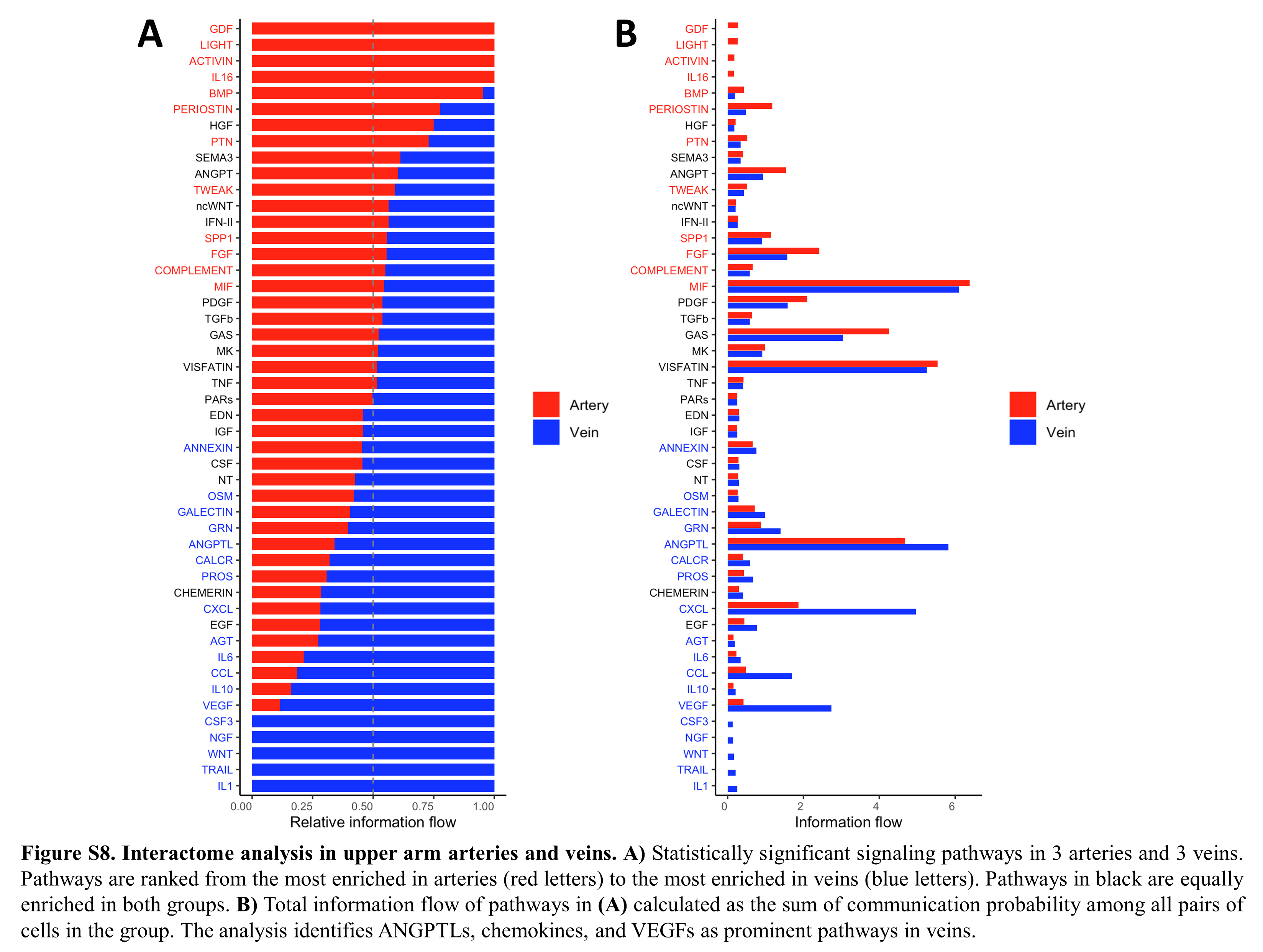

Supplement: Supplementary file 1 [file cells-13-00793-s001.zip › Figure S8.PNG]

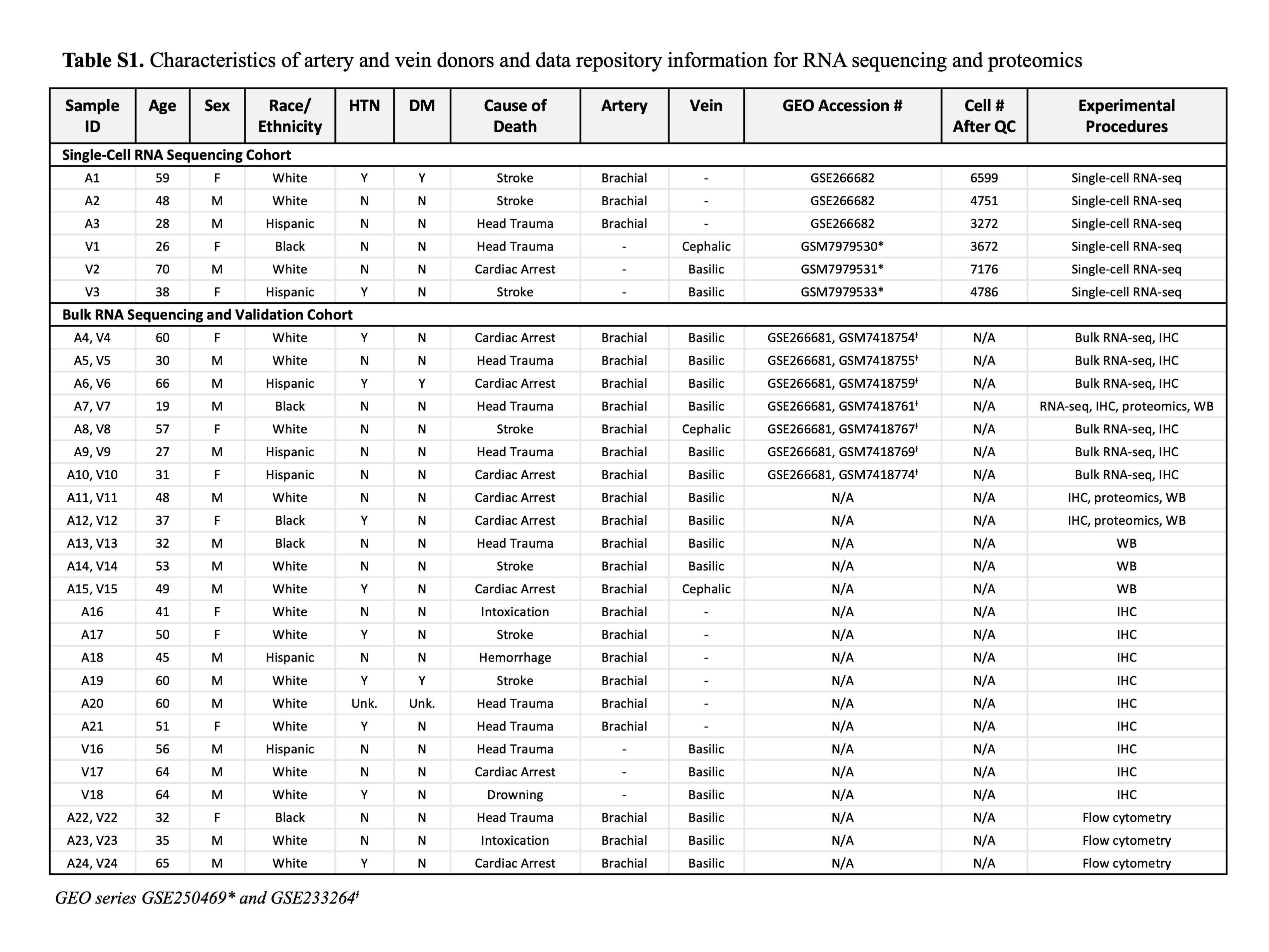

Supplement: Supplementary file 1 [file cells-13-00793-s001.zip › Table S1.png]
